# Decrease in *thyroid adenoma associated (THADA) *expression is a marker of dedifferentiation of thyroid tissue

**DOI:** 10.1186/1472-6890-11-13

**Published:** 2011-11-04

**Authors:** Lars Kloth, Gazanfer Belge, Käte Burchardt, Siegfried Loeschke, Werner Wosniok, Xin Fu, Rolf Nimzyk, Salah A Mohamed, Norbert Drieschner, Volkhard Rippe, Jörn Bullerdiek

**Affiliations:** 1 Center for Human Genetics, University of Bremen, Leobener Str. ZHG, 28359 Bremen, Germany; 2 Department of Pathology, Clinical Center Bremen-Mitte, St. Jürgen Str. 1, 28177 Bremen, Germany; 3 Institute of Statistics, University of Bremen, Achterstr. 30, 28359 Bremen, Germany; 4 Department of Cardiac Surgery, University Medical Center Schleswig-Holstein, Campus Lübeck, Ratzeburger Allee 160, 23538 Lübeck, Germany

## Abstract

**Background:**

*Thyroid adenoma associated (THADA) *has been identified as the target gene affected by chromosome 2p21 translocations in thyroid adenomas, but the role of THADA in the thyroid is still elusive. The aim of this study was to quantify *THADA *gene expression in normal tissues and in thyroid hyper- and neoplasias, using real-time PCR.

**Methods:**

For the analysis *THADA *and 18S rRNA gene expression assays were performed on 34 normal tissue samples, including thyroid, salivary gland, heart, endometrium, myometrium, lung, blood, and adipose tissue as well as on 85 thyroid hyper- and neoplasias, including three adenomas with a 2p21 translocation. In addition, *NIS *(*sodium-iodide symporter*) gene expression was measured on 34 of the pathological thyroid samples.

**Results:**

Results illustrated that *THADA *expression in normal thyroid tissue was significantly higher (*p *< 0.0001, exact Wilcoxon test) than in the other tissues. Significant differences were also found between non-malignant pathological thyroid samples (goiters and adenomas) and malignant tumors (*p *< 0.001, Wilcoxon test, t approximation), anaplastic carcinomas (ATCs) and all other samples and also between ATCs and all other malignant tumors (*p *< 0.05, Wilcoxon test, t approximation). Furthermore, in thyroid tumors *THADA *mRNA expression was found to be inversely correlated with *HMGA2 *mRNA. *HMGA2 *expression was recently identified as a marker revealing malignant transformation of thyroid follicular tumors. A correlation between *THADA *and *NIS *has also been found in thyroid normal tissue and malignant tumors.

**Conclusions:**

The results suggest *THADA *being a marker of dedifferentiation of thyroid tissue.

## Background

Benign thyroid tumors and hyperplasias of follicular epithelial origin belong to the cytogenetically best analyzed human epithelial tumors.

Cytogenetic aberrations have been detected in approximately 20% of these lesions [1]. Translocations of chromosomal band 2p21 are the second most frequent structural chromosomal rearrangement, representing a particular cytogenetic subgroup [2]. The target gene has been identified and referred to as *thyroid adenoma associated *(*THADA*) [3].

The full length cDNA of *THADA *consists of 6,134 bp distributed over 38 exons [GenBank: NM_022065]. There are two splice-variants, one lacking exons 27 and 28 [3], and the other without exons 16 and 17. The THADA protein has three isoforms corresponding to the three different transcript variants with 1953 [GenBank: NP_071348], 1879, and 1832 amino acids, respectively. In adenomas with 2p21 translocations Rippe *et al*. found different types of fusion variants of *THADA *[3]. In each case, *THADA *was truncated after exon 28 and ectopic sequences fused to it were not correlated to any known gene. Thus, it has been speculated that the truncation rather than the fusion to ectopic coding sequences is the critical event for the development of the tumor [3].

Studies by Drieschner *et al*. [4] revealed that the mRNA, the protein size, and the genomic organization is conserved among *Homo sapiens*, *Canis familiaris*, *Chlorocebus aethiops*, *Gallus gallus*, and *Mus musculus*. THADA proteins from the analyzed organisms showed significant assignments to the superfamily ARM repeat (SSF48371; Hidden Markov Models Superfamily database), indicating the presence of a protein-protein-interaction-domain of that type.

The exact function of THADA still remains unclear. Hypothetically, it belongs to the death receptor-interacting proteins and is assumed to bind to death receptor DR5 (Puduvalli VK and Ridgway L, GenBank accession reference note), involving it in the TRAIL-induced apoptosis. The truncated THADA derived from the rearranged allele might compete with the gene product of the normal allele thereby disturbing normal apoptosis of follicular cells, and subsequently altering the steady state between proliferation and cellular death leading to adenomatous growth in benign thyroid tumors with 2p21 translocations [3]. Nevertheless, there is a need for further studies elucidating the role of THADA in normal thyroid development and in tumorigenesis.

Recently, a *THADA *variant has also been linked to type 2 diabetes (T2D) [5], but this association has not been confirmed by the majority of further studies [6-20]. During a meta-analysis of three genome-wide association studies with individuals of European descent Zeggini *et al*. found evidence for an association of a SNP (rs7578597) in exon 24 of *THADA *and the susceptibility for T2D [5]. Further indication for a correlation between *THADA *and T2D was presented in several other publications [11,14,16,17,19], one reported an altered expression of *THADA *in pancreatic islets, using data from the Diabetes Genome Anatomy Project (DGAP) database [11]. In other investigations no correlation was detected [6-8,10,12,13,15,18,20], except for one publication [9], which reported an association between *THADA *SNP rs7578597 and a 2-h insulin level during an oral glucose tolerance test but no significant association between the *THADA *SNP and T2D risk, rendering the association disputable.

The aim of this study was to analyze *THADA *expression in thyroid tissue in comparison to other tissues and to thyroid hyper- and neoplasias to elucidate the possible correlation of *THADA *mRNA with thyroid differentiation and neoplastic growth.

## Methods

### Tissue specimen and RNA isolation

RNA from snap-frozen tissues was isolated using the RNeasy Mini Kit and RNeasy Lipid Tissue Mini Kit for the adipose tissue samples, respectively (QIAGEN, Hilden, Germany).

For the formalin-fixed paraffin-embedded (FFPE) tissues of thyroid tumors, histopathologic diagnoses were performed according to the World Health Organization Classification of Tumours [21] (table 1). As to RNA isolation, FFPE blocks were cut into six sections of 5 μm for each sample using a microtome. Total RNA isolations were performed using the Roche High Pure RNA Paraffin Kit (Roche, Mannheim, Germany) for the *THADA *expression investigation and the RNeasy FFPE Kit (QIAGEN, Hilden, Germany) for the *NIS *expression analysis. Three samples were cytogenetically characterized by 2p21 translocations. In all three cases, two of which published previously [22,23], the breakpoints were narrowed down to the *THADA *locus. One of the anaplastic thyroid samples served as the source of a newly established cell line. Cytogenetical analysis revealed a highly complex karyotype with a range of 80 to 117 chromosomes (100.8 on average). Several marker chromosomes, telomeric associations, and double minutes were detected.

**Table 1:** Histology of the malignant thyroid lesions.

**case no**.	age (years)	sex	histology	tumor diameter (cm)	TNM classification and grading
1	57	f	PTC	0.9	pT1
2	31	m	PTC	2.5	pT2 pN0
3	30	f	PTC	2.5	pT2 NX
4	85	m	PTC	4.0	pT3a
5	31	m	PTC	2.0	pT3 pN1
6	54	f	PTC	0.6	pT1 pNX pMX
7	49	f	PTC	1.2	pT2
8	38	f	PTC	0.6	pT1
9	50	f	PTC	2.2	pT2
10	21	f	PTC	1.0	pT1 pNX pMX
11	38	m	PTC	0.8	pT1; G1
12	34	f	PTC	2.3	pT2 pN1 pMX
13	66	f	PTC	2.0	pT3; G2
14	25	f	PTC	2.3	pT2 pN0
15	42	m	PTC	0.7	pT1 N0 MX
16	42	f	PTC	1.4	pT2a; G2
17	72	f	PTC	1.0	pT1
18	84	f	PTC	6.0	pT3 pNX
19	27	m	PTC	2.5	pT2
20	35	f	FTC	2.1	pT2 pN0 MX
21	66	f	FTC	2.0	pT1
22	67	m	FTC	5.5	pT3 pNX pM1
23	61	m	FTC	8.0	pT4
24	53	f	FTC		pT4 pN1
25	61	m	MTC	3.5	pT2 pN0
26	61	m	MTC	1.7	pT2
27	52	m	MTC	3.3	pT2
28	55	f	MTC	2.2	pT2
29	76	f	ATC	1.7	pT4b
30	76	f	ATC	3.8	pT4b
31	86	f	ATC	9.0	pT4 pN1b pM1
32	65	f	ATC	2.0	pT4 N0; G4

All listed samples were used for the *THADA *expression investigation, for the *NIS *expression analysis samples 2, 3, 7, 13 and 24-28 were omitted. (PTC: papillary thyroid carcinoma; FTC: follicular thyroid carcinoma; MTC: medullary thyroid carcinoma; ATC: anaplastic thyroid carcinoma)

### cDNA-synthesis and real-time PCR expression analysis

RNAs were reverse-transcribed into cDNA by M-MLV Reverse Transcriptase (Invitrogen, Karlsruhe, Germany). Real-time PCR was performed using the Applied Biosystems 7300 sequence detection system according to TaqMan Gene Expression Assay Protocol (Applied Biosystems, Darmstadt, Germany) in 96-well microtiter plates with a total volume of 20 μl. In case of TaqMan gene expression assay of *THADA *(assay number Hs00152982, Applied Biosystems, Foster City, USA), targeting exons 31-32, and of *NIS *(assay number Hs00166567_m1), each reaction consisted of 2 μl of cDNA reverse transcribed from 25 ng of total RNA, 10 μl of TaqMan Universal PCR Master Mix (Applied Biosystems), 1 μl of TaqMan assay and 7 μl of ddH_2_O. For the 18S rRNA assay, using 18S forward and 18S rev_1 primers [24], each reaction consisted of 2 μl of cDNA (1:10 diluted, with regard to higher expression of 18S rRNA) reverse transcribed from 25 ng of total RNA, 10 μl of TaqMan Universal PCR Master Mix, 600 nM of forward and reverse primers, 200 nM of 18S probe [24] and 5.4 μl of ddH_2_O.

Thermal cycling conditions were 2 min at 50°C followed by 10 min at 95°C, 50 cycles at 95°C for 15 s and 60°C for 1 min. A non-template control of amplification and two previous negative controls of cDNA synthesis (one without RNA and one missing Reverse Transcriptase) were included in each plate. Software Sequence Detection Software 1.2.3 (Applied Biosystems) was programmed with the reaction condition. All testing reactions were performed in triplicate.

Serial dilutions were made using cDNA derived from 25, 5, 1, 0.2, and 0.04 ng of total RNA from FFPE tissue of one thyroid adenoma for *THADA *and 18S rRNA, and from fresh frozen tissue of one normal thyroid sample for *NIS*. In each dilution, *THADA*, *NIS*, and 18S rRNA gene expression assays were performed using absolute quantification. Afterwards, the standard curves for both assays were plotted with the log ng of input cDNA for each dilution on the x-axis, and the matched C_T _value on the y-axis. Furthermore, in order to evaluate the differences of amplification efficiencies, the difference of two curve slopes was calculated. If the absolute difference of the slopes is less than 0.1, the amplification efficiencies of two assays are considered to be equal and the comparative C_T _method is valid (User Bulletin No. 2, ABI PRISM 7700 Sequence Detection System, Applied Biosystems). 18S rRNA was used as endogenous control as suggested previously [25-28]. The 18S rRNA assay showed an amplification efficiency of 92.6% (slope = -3.514, R^2 ^= 0.995). The *THADA *assay had an amplification efficiency of 92.0% (slope = -3.531) and an R^2^-value of 0.96. For *NIS*, the amplification efficiency was 93.4% (slope = -3.4917), the coefficient of determination amounted to 0.997). As recommended for FFPE samples [24,29-31] the fragment sizes amplified by all three assays were small, ranging between 60 and 78 bp, a validation of these values was performed via gelelectrophoresis of the PCR-products (data not shown). When applying the comparative C_T _method, one histological normal thyroid tissue was used as calibrator sample. Afterwards, data were compared with results from conventional histology.

For statistical analysis, the Wilcoxon signed rank test was used to compare average values (two-sided, exact version for at most 40 cases involved, otherwise using the t approximation); relationships were quantified by linear regression and Spearman's rank correlation coefficient. Sensitivity, specificity and decision limits were calculated from non-parametric density estimations. Therefore, sensitivity and specificity may differ from raw empirical values and decision limits need not coincide with measured values. A *p*-value of less than 0.05 was considered significant.

### Ethics Statement

The use of human thyroid samples for this study was approved by the local medical ethics committee (Ethikkommission bei der Ärztekammer Bremen) and followed the guidelines of the declaration of Helsinki. Only samples that were initially taken for diagnostic purposes were secondarily used for the present study. During pathological examination, a sample of the tissue was snap-frozen. The procedure was approved by the local ethics committee. Because the samples were deidentified and were considered as samples normally discarded, the committee felt that there was no specific patient consent necessary.

As for the normal tissue samples, these were anonymously collected for earlier studies, each following the guidelines of the declaration of Helsinki.

## Results

### *THADA *expression in normal tissues

Thirty-four snap-frozen samples from eight different tissues were tested for the level of *THADA *expression.

**Figure 1:**
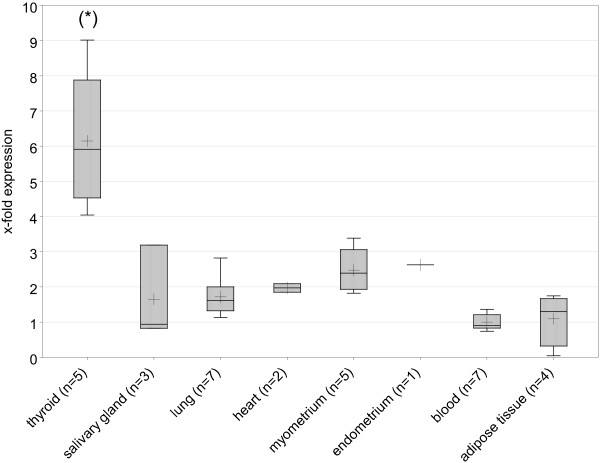
***THADA *expression in normal tissues (snap-frozen samples)**. Boxplots for the relative quantifications of *THADA *gene expression in normal tissues; tissue type at x-axis. (*): *p *< 0.0001 compared to all other tissues jointly (exact Wilcoxon signed rank test). Boxes contain the inner 50% of all values and a bar at the position of the median, whiskers extend to the extrema of values or to 1.5 * box height, whichever is smaller. The plus sign shows the arithmetic mean. (n: number of samples).

The mean level per tissue type ranged from 1 (blood) to 6.14 (thyroid), and the lowest single value for a thyroid sample (4.04) was above the highest one (3.39, myometrium) from any of the other tissues (Figure 1). Accordingly, statistical analysis using Wilcoxon's exact signed rank showed significant differences between normal thyroid tissues and the group of all other tissues (*p *< 0.0001). Using the *THADA *expression to discriminate between thyroid and non-thyroid tissue, a sensitivity of 82.5%, a specificity of 97.4% and an efficiency of 95.2% with a decision limit value of 4.23 were achieved.

### *THADA *expression in thyroid tumors

**Figure 2:**
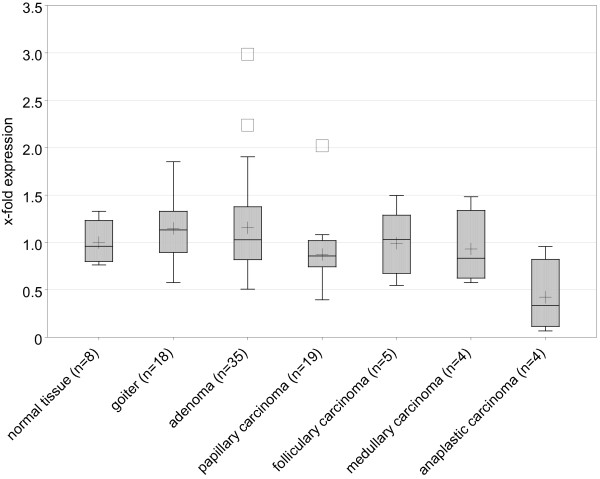
***THADA *expression in thyroid hyper- and neoplasias (FFPE samples)**. Boxplots for the relative quantifications of *THADA *gene expression in thyroid normal tissue, goiter, benign and malignant tumors; normal tissue and hyper-/neoplasia type at x-axis. Boxes contain the inner 50% of all values and a bar at the position of the median, whiskers extend to the extrema of values or to 1.5 * box height, whichever is smaller, isolated symbols indicate values outside this range. The plus sign shows the arithmetic mean. (n: number of samples).

Ninety-three formalin-fixed-paraffin-embedded thyroid samples, including eight normal tissues (from four patients), 18 goiters, 35 benign, and 32 malignant tumors were measured. For single tumor samples the expression ranged between 0.065 (anaplastic carcinoma) and 2.986 (follicular adenoma) in relation to normal tissue, i.e. a ratio of 1 : 45.94. Samples with a 2p21 translocation showed a level of expression of 1.123, 1.624, and 0.662 fold, respectively. The mean values for the different tumor entities ranged from 0.423 (anaplastic carcinoma) to 1.156 (adenoma) (Figure 2 and table 2).

**Table 2:** Detailed view of *THADA *expression in thyroid hyper- and neoplasias

sample type	n	average	standard deviation	median
normal tissue	8	1	0.217	0.959
goiter	18	1.15	0.303	1.132
*nodular goiter*	7	1.266	0.175	1.305
*Graves disease*	1	1.103	-	1.103
*adenomatous goiter*	10	1.073	0.369	1.021
adenoma	35	1.156	0.496	1.029
*autonomous adenoma*	2	0.873	0.212	0.873
*follicular adenoma*	27	1.158	0.522	1.029
*macrofollicular adenoma*	1	1.904	-	1.904
*microfollicular adenoma*	4	1.225	0.268	1.199
*oncocytic adenoma*	1	0.637	-	0.637
carcinoma	32	0.842	0.381	0.842
*papillary carcinoma*	19	0.872	0.352	0.858
*follicular carcinoma*	5	0.991	0.353	1.031
*medullary carcinoma*	4	0.932	0.391	0.834
*anaplastic carcinoma*	4	0.423	0.383	0.334

The arithmetic mean (with the standard deviation) and median relative quantification of *THADA *gene expression in thyroid normal tissue, hyper- and neoplasias are listed. (n: number of samples)

Significant differences of *THADA *expression were noted between benign and malignant thyroid lesions. Wilcoxon's signed rank test showed a highly significant difference comparing the joint group of goiters and benign tumors with malignant tumors (*p *= 0.0009).

Using the exact Wilcoxon test, no significant differences were detected comparing the level of *THADA *expression between normal tissue and benign lesions (*p *= 0.2802) and papillary carcinomas (*p *= 0.2170). In contrast, significant differences were found between anaplastic carcinomas (ATCs), the most dedifferentiated type of thyroid tumors, and all other samples (*p *= 0.0107) and ATCs and all other malignant tumors (*p *= 0.0234). Comparing anaplastic carcinomas with each single group, the difference in expression between ATCs and goiters (*p *= 0.0049) and adenomas (*p *= 0.0058) were marked as significant. As this finding was a result of systematically comparing anaplastic carcinomas with the other lesions, a Bonferroni correction for multiple testing was used (corrected α = 0.0083). Without the need of correcting for multiple testing also normal tissue and papillary carcinoma would have been assessed as significantly different from anaplastic carcinoma (*p *= 0.0485 and *p *= 0.0350, respectively). Overall, significant results were mostly seen with the group of anaplastic carcinomas, indicating a relative stable level of expression in comparatively differentiated tissues with a significant reduction only in dedifferentiated tissues.

Recently *HMGA2 *expression has been shown to indicate thyroid malignancy and can thus be considered marking the dedifferentiation of thyroid epithelium [32-34]. As to the study by Belge *et al*. [32] and the present one 48 samples were identical in both studies (seven normal tissues, one goiter, 15 adenomas and 25 carcinomas, including three anaplastic carcinomas). For these, RNA was isolated from adjacent cuts of the same FFPE block and, except for the different qRT-PCR assays, all samples were treated identical in both investigations. Thus, it was feasible to check these samples for a possible correlation between *THADA *and *HMGA2*. Using Spearman's rank correlation, there was a highly significant inverse correlation between *THADA *and *HMGA2 *expression (correlation coefficient = -0.452; *p *= 0.0015), further underlining a possible role of *THADA *in thyroid differentiation.

NIS (sodium-iodide symporter), the transmembrane glycoprotein accountable for the uptake of iodine in thyroid cells, was found to be a marker of thyroid differentiation [35-38]. To validate our findings *NIS *expression was measured in 41 samples, including seven normal tissue samples, six nodular goiters, five adenomas, and 23 carcinomas (15 papillary, four follicular, and all four anaplastic thyroid carcinomas). Using Spearman's rank correlation, no significant correlation (*p *= 0.1288) was detected comparing *THADA *and *NIS *expression from all samples. By contrast, a significant correlation was found constraining the analysis to the follicular and papillary carcinoma samples (*p *= 0.0497, r = 0.456, n = 19), an even stronger correlation between the expression of *THADA *and *NIS *was found in normal and all malignant samples (*p *= 0.0021, r = 0.540, n = 30), and in normal tissue and anaplastic carcinomas (*p *= 0.0128, r = 0.718, n = 11)

### Transcription factors binding to *THADA*

Using the SABiosciene DECODE Transcription Factor Search, no *THADA*-promotor binding sites for thyroid-specific transcription factors paired box gene 8 (pax8), thyroid transcription factor 1 (TTF1), also known as NK2 homeobox 1 (NKX2-1), and thyroid transcription factor (TTF-2), sometimes referred to as forkhead box protein E1 (FOXE1), were found. Amongst others cAMP response element-binding protein (CREB), activating transcription factor (ATF-2), c-Jun, hepatic leukemia factor (Hlf), and germ cell nuclear factor (GCNF) were marked as relevant, FOXC1, Nkx2-2, Nkx2-5, and Nkx6-1 were displayed with low relevance (data not shown). *HHEX *(hematopoietically expressed homeobox) has been found to be expressed in the adult thyroid gland and in differentiated thyroid cell lines and to be correlated with thyroid differentiation [39-41], but is not included in the SABiosciene DECODE Transcription Factor Search. A manual search for this transcription factor revealed no assured binding sites in the *THADA *promoter.

## Discussion

In this study, *THADA *turned out to be highly expressed in the thyroid compared to other normal tissues. In a group of eight different types of tissue thyroid samples showed a significantly higher *THADA *mRNA expression than salivary gland, lung, heart, myometrium, endometrium, blood, and adipose tissue, hinting at a possibly important role of *THADA *in the thyroid.

The results in part contradict data available online. NCBI ESTProfileViewer predicted a higher expression in heart and lung tissue and a slightly lower in the thyroid. For uterus and blood the data are in concordance with those obtained from the EST-based estimates. For salivary gland and adipose tissue the TPM (transcripts per million)-values are zero, this could be due to an overall small EST pool (20155 ESTs for salivary gland, 13106 ESTs for adipose tissue), resulting in less than one gene EST (all normal tissues average: 31073 ESTs per gene EST). Comparison to Affymetrix GeneChip Human Genome array-based results from The Genomics Institute of the Novartis Research Foundation (GNF) showed similar discrepancies. There are three probes, one (gnf1h10751_at) is diverging considerably from the other two and was therefore omitted. Compared to our data both remaining probes resulted in similarly average Spearman's rank correlation coefficients and no significances (*p *≥ 0.2). GNF results showed thyroid as the tissue with the highest *THADA *expression but less distinct from the other tissues. Overall, the more precise and reliable qRT-PCR-method disclosed results that are diverging from those available from online databases.

Furthermore, evidence that *THADA *expression is associated to thyroid differentiation has been presented. Analysis of 93 thyroid FFPE samples revealed significant differences between benign and malignant thyroid lesions, especially when comparing the group of anaplastic carcinomas with other types of lesions. Despite one outlier with an expression level almost identical to normal tissue, the values were significantly lower compared to all other samples as well as to all other malignant tumors. A comparison of the expression level of *THADA *and *NIS *(*sodium-iodide symporter*) confirmed these observations. Amongst others, a significant correlation between *THADA *and this well established marker of thyroid differentiation [35-38] has been detected in normal tissue and anaplastic carcinomas. This suggests that *THADA *expression decreases with dedifferentiation of the thyroid epithelium. This hypothesis is further supported by the significant inverse correlation between the expression of *THADA *and *HMGA2*. Belge *et al*. [32] showed that *HMGA2 *is significantly overexpressed in malignant thyroid tumors compared to benign lesions. As a rule, a high *HMGA2 *expression seems to be accompanied by a low *THADA *expression. As yet the underlying mechanism is unknown but it does not seem to involve thyroid-specific transcription factors, since no binding sites for pax8, TTF-1 and -2 were found. However, the SABiosciene DECODE Transcription Factor Search revealed a binding site of the cAMP response element-binding protein (CREB). CREB has been shown to regulate diverse cellular responses, including differentiation [42], targeted expression of dominant-negative mutants of CREB in transgenic mice has been associated with thyroid hypoplasia [43]. cAMP indirectly plays a crucial role in the differentiation of endocrine tissues [43], including the thyroid [44,45]. Thus one might speculate about an involvement in the decreased expression of *THADA *in dedifferentiated thyroid cells.

In thyroid adenomas *THADA *was frequently found to be truncated [3]. Whereas the intact *THADA *may be involved in maintaining the differentiation of thyroid epithelium, the truncated allele might play a key role in tumor development of the thyroid. While competing with the full-length protein translated from the normal allele of *THADA *the altered protein derived from the truncated gene might lead to an impaired induction of apoptosis, and subsequently give rise to an increased cell proliferation leading to benign thyroid tumors with 2p21 translocations [3], without significant changes of the expression level.

## Conclusions

*THADA *expression, though not restricted to the follicular cells of the thyroid, is higher in the thyroid than in other tissues tested (salivary gland, heart, endometrium, myometrium, lung, blood, and adipose tissue). As to its normal function, *THADA *expression has been found to be decreased in anaplastic carcinomas and to be correlated with the expression of *NIS*, a marker of thyroid differentiation, and inversely correlated with that of *HMGA2*, a marker of malignant transformation of the thyroid and cancer stemness. It may thus have essential functions in maintaining the differentiation of the follicular epithelium.

## Declaration of competing interests

The authors declare that they have no competing interests.

## Authors' contributions

LK conceived and designed the study, carried out the molecular genetic studies, took part in the statistical analysis and the search for transcription factors and drafted the manuscript. GB provided the study material (FFPE and part of the snap-frozen samples), and revised the manuscript. KB carried out the clinical workout and performed the pathological analysis. SL performed the pathological determination of the samples (verification). WW carried out the statistical analysis. XF took part in the molecular studies. RN took part in the search for transcription factors. SM provided the study material (part of the snap-frozen samples) and took part in the analysis and interpretation of the obtained data. ND provided background information of certain samples and took part in the analysis and interpretation of the obtained data. VR took part in the analysis and interpretation of the obtained data. JB conceived the study and participated in its design and coordination and helped to draft and revised the manuscript. All authors have read and approved the final manuscript

## Pre-publication history

The pre-publication history for this paper can be accessed here:

http://www.biomedcentral.com/1472-6890/11/13/prepub
